# Supplementary material for: Dnmt3a2/Dnmt3L Overexpression in the Dopaminergic System of Mice Increases Exercise Behavior through Signaling Changes in the Hypothalamus
Source: Int J Mol Sci. 2020 Aug 31;21(17):6297. doi: 10.3390/ijms21176297 (PMC7504350; doi:10.3390/ijms21176297)
Supplement: Supplementary file 1 [file ijms-21-06297-s001.pdf]

International Journal of
Molecular Sciences

Supplemental Figures

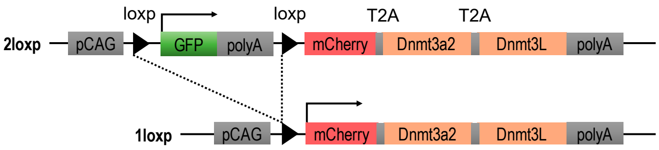

Figure S1. Schematic representation of the Cre/loxp system strategy. 2loxp vector (upper) transcribes only GFP, but not the following sequences. When crossed with Dat cre, the GFP cassette between two loxp sites will be removed, leaving the 1loxp allel (lower). In the 1loxp vector, mCherry and Dnmt3a2 as well as Dnmt3L start to transcribe. The sequences connecting mCherry, Dnmt3a2 and Dnmt3L are T2A peptides.

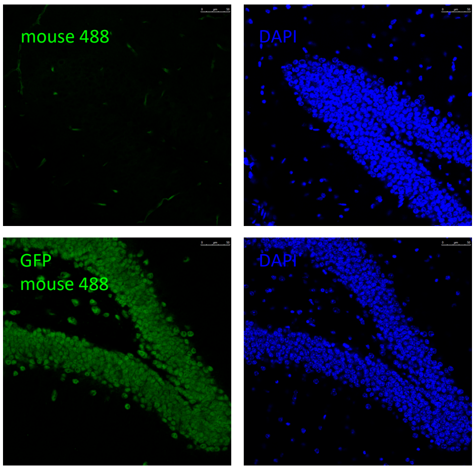

Figure S2. Endogenous GPF without anti-GFP staining (upper left) was almost undetectable.

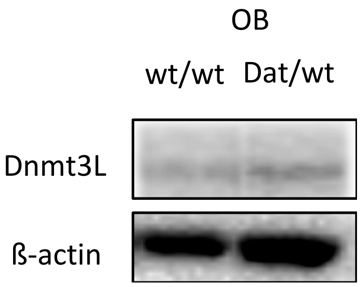

Figure S3. Dnmt3L can only be detected in OB.

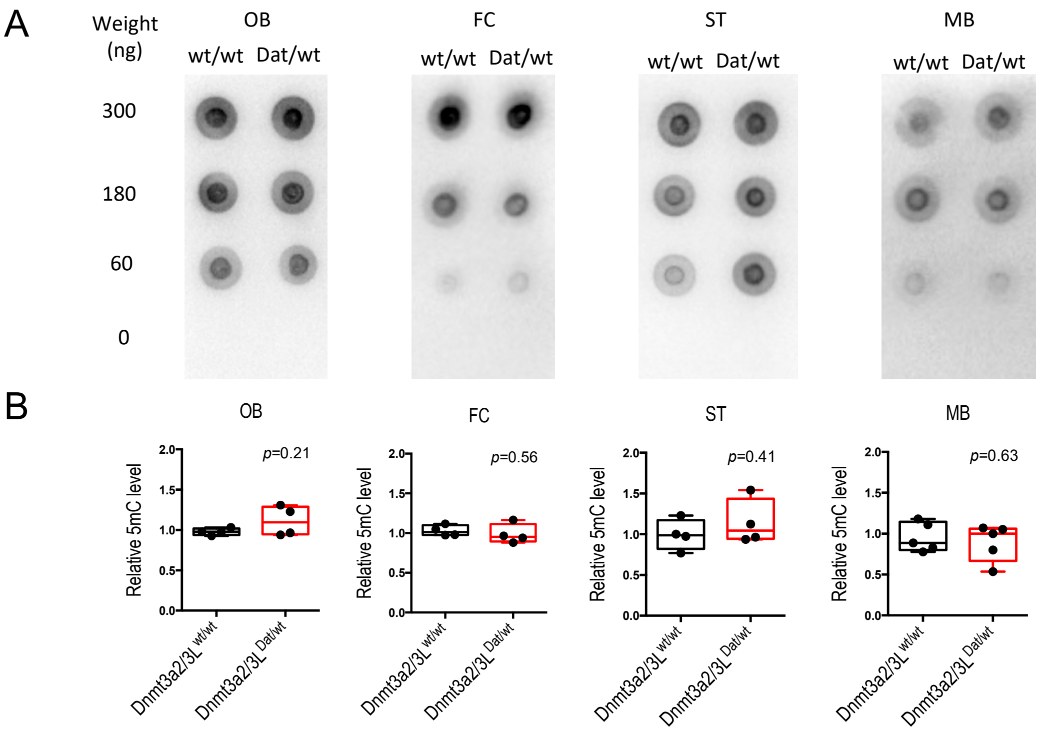

Figure S4. Dot blot of 5mc on tissue level was shown no significant difference in Dnmt3a2/3L$^{wt/wt}$ and Dnmt3a2/3L $^{Dat/wt}$ animals. (**A**) Dot blot of 5mc from 0ng, 60ng, 180ng, 300ng protein from olfactory bulb (OB), frontal cortex (FC), striatum (ST) and mid brain (MB). (**B**) Quantification of 5mc from dot blot of 180ng protein groups. n = 4, unpaired t test.

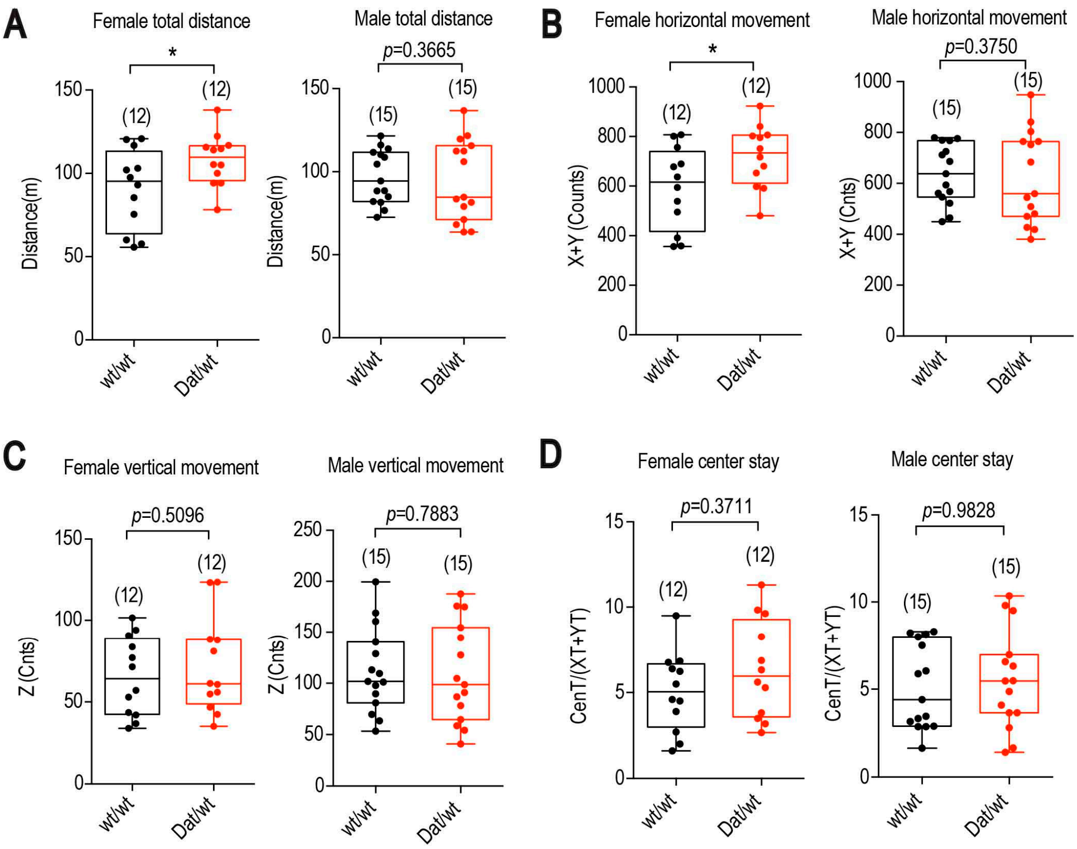

Figure S5. Dnmt3a2/3L$^{Dat/wt}$ female mice showed increased spontaneous motor activities. (**A**,**B**) In the female group, the overall activity, including distance and horizontal movement of Dnmt3a2/3L$^{Dat/wt}$ mice was increased significantly compared to control littermates by 15–20%. No difference was

observed in the male group. (**C**) Vertical movements of Dnmt3a2/3L$^{Dat/wt}$ mice showed no significant differences in any group. (**D**) Time spent in the centre area also showed no difference in all groups. Values are reported as the mean ± SEM. * p < 0.05. Student's t-test for comparison between Dnmt3a2/3L$^{wt/wt}$ and Dnmt3a2/3L$^{Dat/wt}$ of each gender.

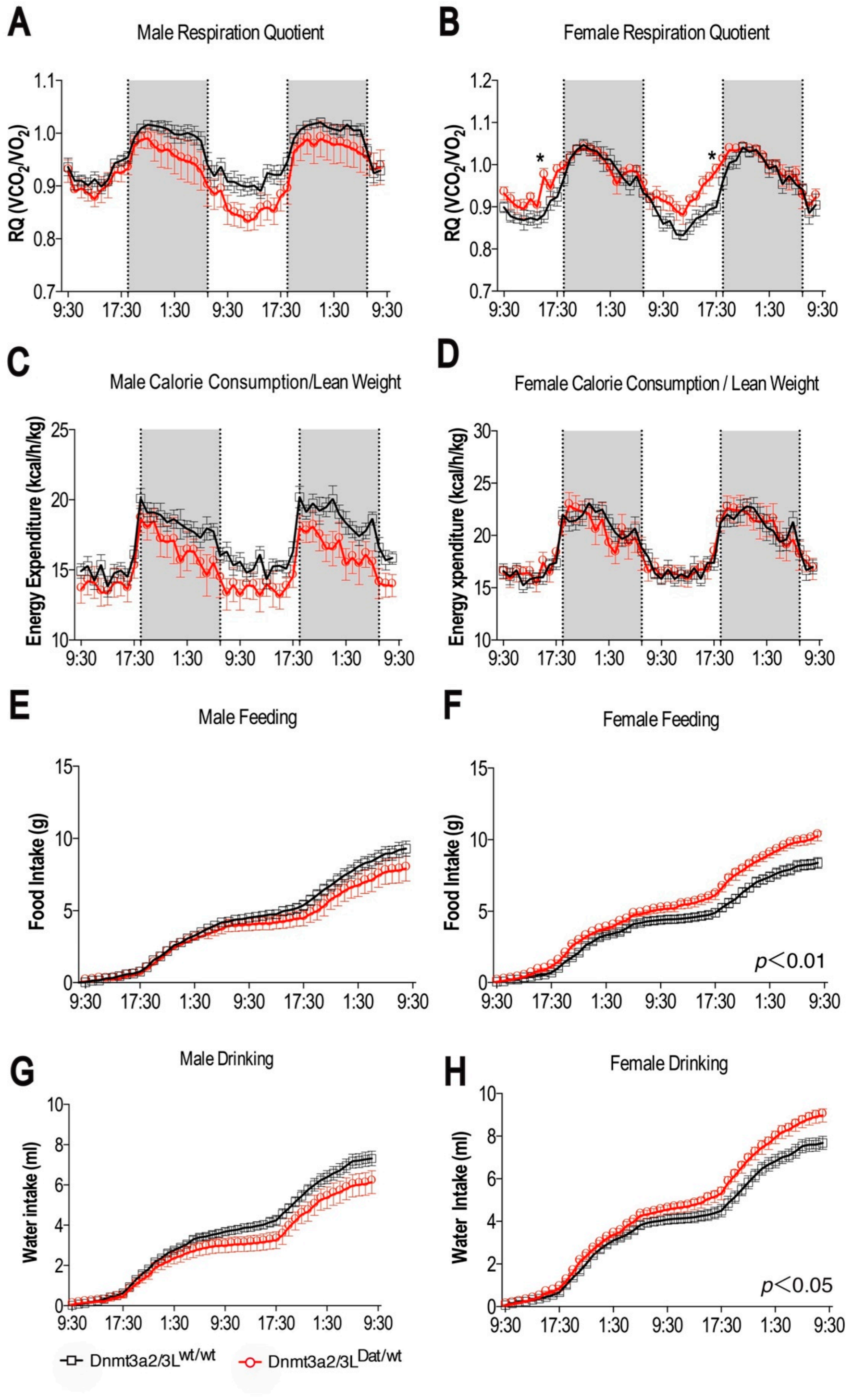

Figure S6. Dnmt3a2/3L$^{Dat/wt}$ mice showed the normal metabolism and the higher food and drinking intake. (**A**,**B**) Dnmt3a2/3L$^{Dat/wt}$ females showed higher respiration quotient in daytime. Dnmt3a2/3L$^{Dat/wt}$ males tended to have lower respiration quotient without significance. (**C**,**D**) Dnmt3a2/3L$^{Dat/wt}$ females showed the same energy consumption as Dnmt3a2/3L$^{wt/wt}$ group, Dnmt3a2/3L$^{Dat/wt}$ males tended to have lower respiration quotient without significance. (**E**–**H**) Dnmt3a2/3L$^{Dat/wt}$ females exhibited significant higher food and water intake compared to

Dnmt3a2/3L$^{wt/wt}$ group, while males group showed no difference. 2-way anova with multiple comparisons test (Bonferroni used for correction) was used, * adjusted $p < 0.05$.

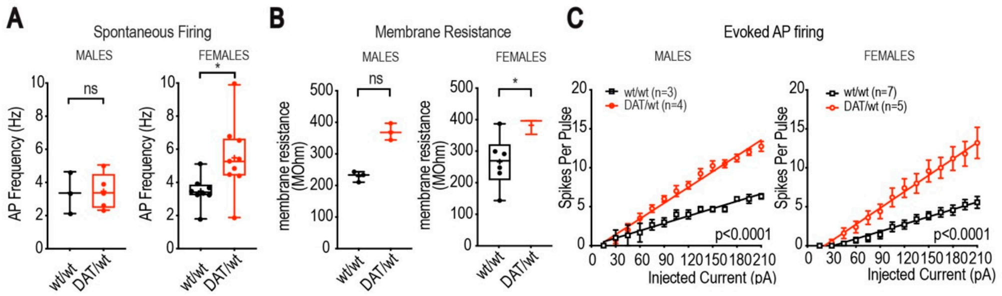

Figure S7. Spontaneous activity and neuronal excitability were increased significantly in female Dnmt3a2/3L$^{Dat/wt}$ animals, and shown the same tendencies in males. (**A**) Spontaneous firing of dopaminergic neurons was significantly increased in female Dnmt3a2/3L$^{Dat/wt}$ animals, while shown no difference in the male group. (**B**) Dopaminergic neurons of Dnmt3a2/3L$^{Dat/wt}$ animals shown the same tendencies of high membrane resistance in both male and female groups. (**C**) Neuronal excitability were increased significantly in male and female groups of Dnmt3a2/3L$^{Dat/wt}$ animals. two-tailed Mann-Whitney-Test.

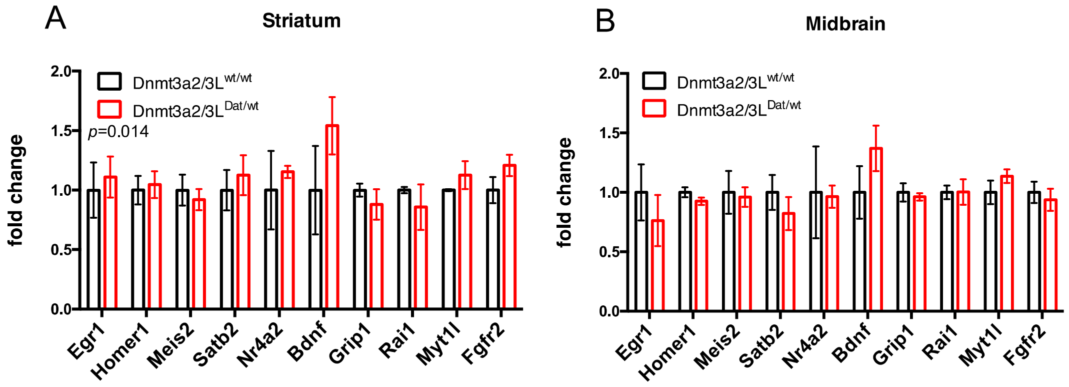

Figure S8. Dnmt3a2/3L over-expression tends to up-regulate Egr1 in striatum Dnmt3a2/3L$^{Dat/wt}$ animals. $n \geq 3$, unpaired t test. Synaptic plasticity gene expression in striatum (**A**) and midbrain (**B**) of Dnmt3a2/3L$^{Dat/wt}$ animals and control littermates.

Supplemental Experimental Procedures

Generation of Dnmt3a2/3L$^{Dat/wt}$ Mice

Flox-Dnmt3a2/3L (C57BL6N) mice were generated through IVF by Transgenesis core-facility in MPI for Biology of Ageing using embryos got from Prof. Obata's lab in Tokyo University of Agriculture [20]. CaMKIIa-cre [56] and Dat-cre [21] were received from Prof. Larsson´s lab in MPI for Biology of Ageing, previously generated in-house. To avoid the leaking effect of Dat-cre females, which will cause the whole-body over-expression, we used male Dat cre/+ and female Flox-Dnmt3a2/3L animals to generate Dnmt3a2/3L$^{Dat/wt}$ mouse line.

Genotyping

Ear notches from ear tagged mice were lysed using 75 μL quick extraction Buffer I at 96 degrees for 45 min, and then the samples were cooled down on ice for 5 min, before adding 75 μL Buffer II. Mix well by vortex before used for genotyping PCR. The primers were used for the genotyping were

indicated as Table S1. Amplified DNA was separated using 2% agarose gel and detected by 1% SYBR Safe DNA gel stain (Invitrogen, S33102) and visualized using Gel Imaging G-Box. Buffers I and II were stored at room temperature in 10 times stock, and diluted to working concentration before use, and the components are as below:

10 × Buffer I: 5 g NaOH, 0.3722 g EDTA, 500 mL mqH_2O

10 × Buffer II: 200 mL 1M Tris HCL(pH = 8), 500 mL mqH_2O

Table S1. Primers were used for genotyping in this study.

Name	Sequence	Annealing Temperature (°C)	PCR Product Size (bp)	
			Positive	WT
EGFP-F	tgaa ccgc atcg agct gaa ggg	66	300	N/A
EGFP-R	tcca gcag gacc atgt gat cgc			
Dnmt3a2-F	ggac aaga atgc tacc aaag	56	425/189	425
Dnmt3a2-R	catc tccg aacc acat gacc			
Dnmt3L-F	cggg agac acct tctt cttg	59	561/167	561
Dnmt3L-R	ggct cttt gcag tctt ccag			
Dat-F	catg gaat ttca ggtg cttgg	50	470/310	310
Dat-R	cgcg aaca tctt cagg ttct			

Immunohistochemistry

Brains from Dnmt3a2/3L$^{Dat/wt}$ mice and Dnmt3a2/3L$^{wt/wt}$ littermate controls were harvested, fixed in 4% PFA for 24 h, and dehydrated in 20% and 30% sucrose for 24 h until they sank, respectively. Brain was embedded in OCT and middle horizontal sections (30 µm) were incubated in PBS supplement with 5% donkey serum for one-hour blocking. The slices were immunostained with primary antibody or in blocking buffer as negative control overnight at 4 °C. The next day, the slices were washed with PBS (6 × 5 min), and incubated with secondary antibody for 2 h at room temperature, washed again with PBS (6 × 5 min) and mounted using mounting gel (Thermo Fisher, P36965, Waltham, MA, USA). The brain slices were visualized under fluorescence microscope.

For DAB staining of mCherry reporter, first antibody anti-RFP (Rockland) was used. The second day after washing, the brain sections were incubated in the secondary antibody of HRP reagent for 1 h. Reactions were visualized by developing DAB substrates under a light microscope.

Genomic DNA Extraction and Dot Blot

Genomic DNA was extracted using DNeasy Blood & Tissue Kit (Qiagen, Hilden, Germany). Draw grid by pencil on nitrocellulose membrane. 5 µL genome DNA of empty vector control, Dnmt3a2 OE and Dnmt3a2/3L OE were pipetted slowly at the centre of the grid. 0, 60, 180, 300 µg amount of DNA each sample were measured. After the membrane dried completely, 20 min UV radiation was applied to fix the DNA. The membrane was incubated in 5% BSA for 2 h, and then with primary antibody 5mC (ActiveMotif, #39649, 1:2000, Carlbad, CA, USA) overnight at 4 °C, washed with PBST (3 × 5 min), and incubated with secondary rabbit antibody (1:10000) conjugated with HRP in 5% BSA for 2 h at room temperature. The membrane was developed with ECL reagent (Amersham, Little Chalfont, UK). Protein levels were quantified by Image Lab (Bio-Rad). Three independent samples were analyzed.

DNA Methylation Level Measurement

5mC and TH were stained as the immunohistochemistry protocol. The cell fluorescence intensity was measured by ImageJ (http://rsbweb.nih.gov/ij/download.html). Select the TH positive cells using the drawing tools, and select the 'set measurements' for the area of cell body. The selected TH positive cells were automatically circled for each channel including 5mC (Figure S9). The 5mC fluorescence intensity was devided by TH positive signal for normalization in each cell. Take six fields each brain from three animals in Dnmt3a2/3L$^{Dat/wt}$ and Dnmt3a2/3L$^{wt/wt}$ cohorts.

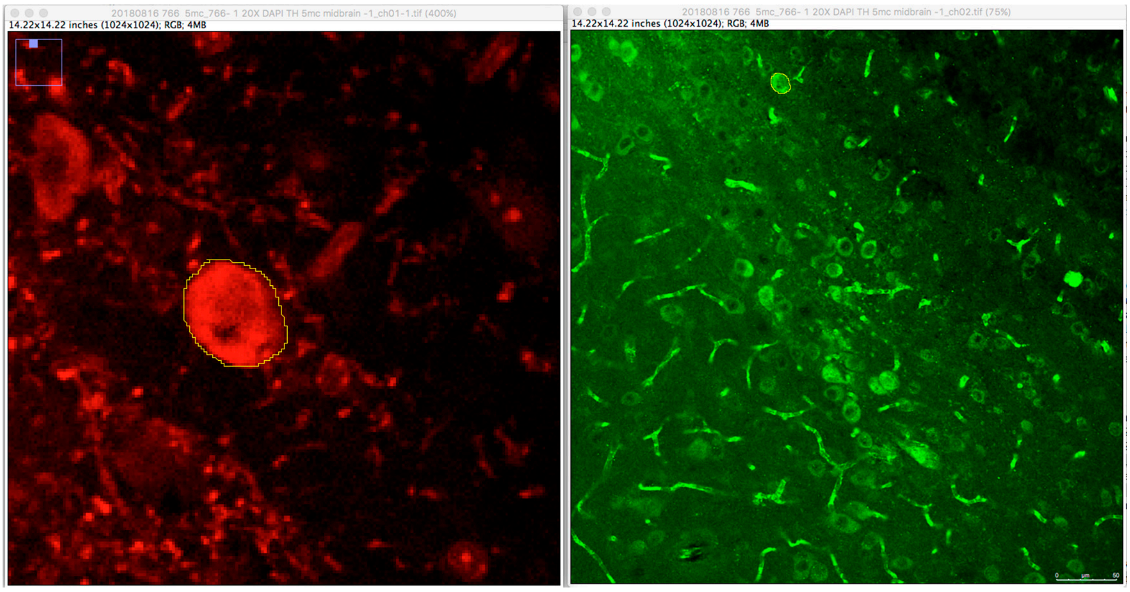

Figure S9. TH positive cell body in RFP channel was selected and measured. 5mC fluorescence from the same selected cell was measured correspondingly.

RNA Extraction and Quantitative PCR

Total RNA was isolated according to TRIZOL reagent protocol (TRIzol™ Reagent 15596026). Reverse transcription kit (Invitrogen) was used for revers transcribing 1 µg total RNA into cDNA. Quantitative RT-PCR was performed on thermal cycler using TaqMan gene expression assays for the target genes (Table S2) Expression levels of target genes were normalized to the expression of the housekeeping gene *GAPDH*.

Table S2. Primers were used for qRT-PCR in this study.

Name	Sequence
GAPDH-F	accacagtccatgccatcac
GAPDH-R	accttgcccacagccttg
Dnmt3a2-mRNA-F	ggaaagatcatgtacgtcgg g
Dnmt3a2-mRNA-R	gccagtaccctcataaagtcc
Dnmt3L-mRNA-F	tctcccactgtctcag gc
Dnmt3L-mRNA-R	tccaggtccaaggtttcaag
Egr1-mRNA-F	agcgccttcaatcctcaag
Egr1-mRNA-R	tttggctgggataactcgtc
Homer1-mRNA-F	cagagcaagttttcattgggc
Homer1-mRNA-R	tgtgttcgggtcaatctgg
Meis2-mRNA-F	ttggcggacaggttatggac
Meis2-mRNA-R	tgcgtgtgtttccttcttcct
Satb2-mRNA-F	caagagtggcattcaaccgc
Satb2-mRNA-R	acgcagtcctgggatcttct
Nr4a2-F	tgttcgcacttgtgaggg
Nr4a2-R	agctaggcacttctgaaacc
Bdnf-mRNA-F	gtgacagtattagcgagtggg
Bdnf-mRNA-R	gggattacacttggtctcgtag
Grip1-mRNA-F	gccaaggaaacttagggaaca
Grip1-mRNA-R	ctctgtggtgcccattcc
Rai1-mRNA-F	tgtaccagctgtcacgagc

Rai1-mRNA-R	gcacttcaaagtaaaattctcctcaat
Myt1l-mRNA-F	ctcgagagagtacctgcagac
Myt1l-mRNA-R	agacccccaaatgtgcagac
Fgfr2-mRNS-F	aaggtttacagcgatgccca
Fgfr2-mRNS-R	accaccatgcaggcgattaa

Immunoblotting

Cells (~2 × 10^6) or brain tissues were lysed in RIPA buffer supplemented with protease and phosphate inhibitors (Roche, Basel, Switzerland). An equal amount of proteins was separated by SDS-PAGE gels (Biorad, Hercules, CA, USA), transferred onto nitrocellulose membrane (GE Healthcare, Chicago, IL, USA) using 100 V 30–45 min, blocked with 5% non-fat milk or BSA in TBST, and immunoblotted with different antibodies (Table S3). The membrane was developed with ECL reagent (Amersham, Little Chalfont, UK). Protein levels were quantified by Image Lab (Bio-Rad, Hercules, CA, USA). Three independent samples were analyzed.

Table S3. Antibodies used in this study.

Antibody	Dilution (Sample Type)	Catalogue
Anti-RFP	1:1000	tebu-bio GmbH, # 039600-401-379
Dnmt3a2	1:1000 (tissues)	Millipore, #07-2050
Dnmt3L	1:1000 (tissues)	cell signaling, #13451
5mC	1:2000 (membrane)<br>1:500 (tissues)	ActiveMotif, #39649
Anti-TH	1:200 (tissues)	Millipore, #AB1542

Metabolic Cages (Phenomaster)

The energy homeostasis of the test animals is analyzed in detail using indirect calorimetry. The measurements are carried out in metabolic cages (Phenomaster, TSE Systems, Bad Homburg, Germany), which correspond to type II housing cages in terms of their base and volume (26.5 cm × 20.5 cm × 14 cm, 543 cm^2, 7.6l m^3) and with litter and Nestlets are provided. Before the actual measurements of the indirect calorimetry, the test animals are prepared for the measurements in so-called exercise cages. In the exercise cages, the animals get used to individual housing, the hanging drinking bottles and the special drinking nipples as well as the hanging feeding racks. During the time (3–4 days) in the exercise cages, the mice were closely monitored for their drinking and eating behavior and weighed daily to ensure that the changed dosage form was accepted. Then, there was a further acclimatization phase of 24 h in the actual metabolic cages. The actual measurement takes four days (48 h basal and 48 h with the impeller). Inside the cages, the mice were supplied with a constant flow of air. Because of their metabolism, the animals consume oxygen and produce carbon dioxide. In indirect calorimetry, the difference between the oxygen and carbon dioxide levels in the air was measured before and after it had been fed into the individual cages. From this difference, the oxygen consumption, carbon dioxide production, the respiratory quotient, and the energy expenditure of the test animals can be determined. In addition to indirect calorimetry, the spontaneous activity of the animals is also documented using light barriers. In addition, the optional activity of the test animals is documented with the help of running wheels. Furthermore, the feed and water consumption of the test animals is measured by automated feed and water weighing sensors.

Keeping the test animals individually is essential for the metabolic measurements. However, after the experiment, the animals can be returned to their original group housing. The animals have the possibility of visual contact during individual housing.

Electrophysiology

Animals and Brain Slice Preparation

Experiments were performed on coronal brain slices from 14–16 weeks old male and female Dnmt3a2/3L$^{Dat/wt}$ mice (n = 3) and Dnmt3a2/3L$^{wt/wt}$ littermate (controls) (n = 3). Animals were lightly anesthetized with isoflurane (B506; AbbVie Deutschland GmbH and Co KG, Ludwigshafen, Germany) and subsequently decapitated. The brain was rapidly removed and a block of tissue containing the mesencephalon was immediately dissected. Coronal slices (250–300 µm) containing the SN were cut with a vibration microtome (HM-650 V; Thermo Scientific, Walldorf, Germany) under cold (4 °C), carbogenated (95% O_2 and 5% CO_2), glycerol-based modified artificial cerebrospinal fluid (GACSF). GACSF contained (in mM): 250 Glycerol, 2.5 KCl, 2 $MgCl_2$, 2 $CaCl_2$, 1.2 NaH_2PO_4, 10 HEPES, 21 $NaHCO_3$, 5 Glucose adjusted to pH 7.2 (with NaOH) resulting in an osmolarity of ~310 mOsm. Brain slices were transferred into carbogenated artificial cerebrospinal fluid (ACSF). First, they were kept for 20 min in a 35 °C 'recovery bath' and then stored at room temperature (24 °C) for at least 30 min prior to recording. ACSF contained (in mM): 125 NaCl, 2.5 KCl, 2 $MgCl_2$, 2 $CaCl_2$, 1.2 NaH_2PO_4, 21 $NaHCO_3$, 10 HEPES, and 5 glucose adjusted to pH 7.2 (with NaOH), resulting in an osmolarity of ~310 mOsm. For the experiments, brain slices were transferred to a recording chamber (~1.5 mL volume) and continuously superfused with carbogenated aCSF at a flow rate of ~2 mL·min^{-1}. Experiments were carried out at ~32 °C using an inline solution heater (SH27B; Warner Instruments, Hamden, CT, USA) operated by a temperature controller (TC-324B; Warner Instruments). Neurons in the SN were visualized with a fixed-stage upright microscope (BX51WI; Olympus, Hamburg. Germany), equipped with a 20× water immersion objective (XLUMplan FI; 20×; 0.95 numerical aperture; Olympus), a 4× magnification changer (U-TVAC, Olympus), and infrared-differential interference contrast [57].

Perforated Patch Recordings

Ventral tier SN DA neurons were identified by the size and location of their somata, and by their electrophysiological fingerprint, i.e., slow and regular firing and the presence of a large I_H (hyperpolarization-activated cation current)—dependent 'sag' potential [58–60]. Recordings were performed with a modified ELC03-XS amplifier (NPI Electronic, Tamm, Germany) controlled by the software PatchMaster (version 2.32; HEKA, Lambrecht, Germany) via a LIH 1600 data acquisition system (HEKA). In Parallel data were sampled at intervals of 20 µs (50 kHz) with a CED 1401 using Spike2 (version 7) (both Cambridge Electronic Design, Cambridge UK) and low-pass filtered at 10 kHz with a four-pole Bessel filter**.** The liquid junction potential between intracellular and extracellular solution was compensated (14.6 mV; calculated with Patcher's Power Tools plug-in for Igor Pro 6 (Wavemetrics, Portland, OR, USA).

Perforated patch recordings were performed using protocols modified from Horn & Marty [61] and Akaike & Harata [62]. Electrodes with tip resistances between 3 and 5 MOhm were fashioned from borosilicate glass (0.86 mm inner diameter; 1.5 mm outer diameter; GB150- 8P; Science Products) with a vertical pipette puller (PP-830; Narishige, London, UK). Patch recordings were performed with ATP and GTP free pipette solution containing (in mM): 128 Kgluconate, 10 KCl, 10 HEPES, 0.1 EGTA, 2 MgCl2 and adjusted to pH 7.3 (with KOH) resulting in an osmolarity of ~300 mOsm. ATP and GTP were omitted from the intracellular solution to prevent uncontrolled permeabilization of the cell membrane [63]. The patch pipette was tip filled with internal solution and back filled with 0.02% tetraethylrhodamine-dextran (D3308, Invitrogen, Eugene, OR, USA) and amphotericin-containing internal solution (~200–250 µg·ml^{-1}; G4888; Sigma-Aldrich, Taufkirchen, Germany) to achieve perforated patch recordings. Amphotericin was dissolved in dimethyl sulfoxide (final concentration: 0.1–0.3%; DMSO; D8418, Sigma-Aldrich, St. Louis, MO, USA) as described previously [64], and was added to the modified pipette solution shortly before use. The used DMSO concentration had no obvious effect on the investigated neurons. During the perforation process, access resistance (Ra) was constantly monitored and experiments were started after Ra and the action potential amplitude were stable (~15–20 min). A change to the whole-cell configuration was indicated by diffusion of tetraethylrhodamine-dextran into the neuron. Such experiments were rejected. To block GABAergic

and glutamatergic synaptic input the aCSF contained 10^{-4} M picrotoxin (P1675; Sigma-Aldrich), 5 × 10^{-5} M D-AP5; A5282; Sigma-Aldrich), and 10^{-5} M CNQX (C127; Sigma-Aldrich, St. Louis, MO, USA). The cell input resistance was calculated from voltage responses to small hyperpolarizing current pulses. To analyze excitability, i.e., evoked action potential firing, a series of depolarizing current pulses (15 pA to 210 pA in 15 pA increments; 1.5 s duration) were injected from a holding potential of −70mV. For each current pulse, the number of action potentials was determined.

Data Analysis

Data analysis was performed with Spike2 (Cambridge Electronics, Cambridge, MA, USA), Graphpad Prism (version 5.0b; Graphpad Software Inc., La Jolla, CA, USA) and Igor Pro 6 (Wavemetrics, Portland, OR, USA). If not stated otherwise, all calculated values are expressed as means ± SEM (standard error of the mean). The '+' signs in the box plots show the mean, the horizontal line the median of the data. The whiskers indicate the smallest and the largest values, respectively. Two-tailed unpaired *t* tests or Welch's unpaired *t* tests were performed to determine differences in electrophysiological properties between SNc DA neurons of Dnmt3a2/3L$^{Dat/wt}$ and Dnmt3a2/3L$^{wt/wt}$ mice. A significance level of 0.05 was accepted for all tests. In the figures *n* values are given in brackets. To characterize the spontaneous activity in each cell, 50 consecutive interspike intervals (ISIs) were chosen, and from these ISIs, the coefficient of variation (CV) was calculated. The intrinsic pacemaking of a DA neuron was regarded as intact if the CV was less than 10% and ISIs were normally distributed (D'Agostino & Pearson normality test).

Metabolite Extraction

Mouse striatum was prepared from Dnmt3a2/3L$^{Dat/wt}$ and Dnmt3a2/3L$^{wt/wt}$ mice. The metabolite extraction was performed as described by [52] with slight modifications as described below. Frozen striatum was homogenized using a Tissue Lyzer (Qiagen, Hilden, Germany). Metabolites were extracted by adding 100 µL of pre-cooled (–20 °C) LC-MS-grade acetonitrile (VWR, Ulm, Germany) and the mixture was vortexed until a homogeneous mixture was obtained. After a 5 min centrifugation step at 4 °C and 20,000× *g*, the supernatant was collected. For the derivatization of the mouse metabolites, as well as the reference compounds, 100 µL of the metabolite extract/reference compounds were mixed with 50 µL of 100 mM sodium carbonate in 0.22µm MilliQ-Water and 50 µL of a 2% benzylchloride solution in acetonitrile. The samples were vortexed for 30 sec and 100 µL of 0.22 µm MilliQ-Water was added. Sample were vortexed again for another 30 sec before being centrifuged again for 5 min at 20,000× *g*. Clear supernatants were transferred to LC-MS glass vials for UPLC-MS analysis.

UPLC-MS Analysis

For the quantitative analysis of metabolites from mouse striatum tissue, samples and related reference compounds (Sigma, St. Louis, Missouri, US) were separated and detected as described in [52] with slight modifications. Sample analysis was performed using an Acquitiy UPLCTM I-class chromatographic system coupled to a XevoTM TQ-S mass spectrometer (Waters, Elstree, UK). The measurments were performed in positive electrospray ionization (ESI) mode using multi reaction monitoring (MRM), employing the following settings for the mass spectrometer: capillary voltage 2.4 kV, cone temperature 150 °C, desolvation temperature 400 °C, desolvation gas flow 800 L/h, cone gas flow 150 L/h, and collision gas flow 0.15 mL/min. The chromatography was performed using an Acquity BEH C18 column with 1.7 µm particles and 2.1mm × 50 mm (diameter × length), using the following solvents and settings: Solvent A: 0.22 µm MilliQ-Water containing 10 mM ammonium (Sigma, St. Louis, Missouri, US) and 0.15% Formic Acid (Biosolve, Valkenswaard, Netherlands). Solvent B: acetonitrile (VWR, Ulm, Germany). The column temperature was 27 °C and the gradient started with 50% A and a flow rate of 0.3 mL/min. After an isocratic step of 1.5 min A was decreased

to 0% using a linear gradient of 5.5 min. This setting was held for another 5 min, before setting back to 50% A within 0.1 min and reequilibrate the column for 2.9 min.

For the selective analysis of the catecholamines from the striatum the following MRM transitions were used: As quantifier (M+H+)+ for Benzoyl-NPP m/z 482.33 to m/z 105.17 with a cone voltage of 46V and collision energy of 20V, Benzoyl-Dopamine (DA) m/z 466.33 to m/z 105.23 with a cone of 60V and collision energy of 18V, Benzoyl- 3,4-Dihydroxyphenylacetic acid (DOPAC) m/z 394.33 to m/z 105.24 with a cone of 38V and collision energy of 18V, Benzoyl- 5-Hydroxytryptophan (5-HT) m/z 385.35 o m/z 105.17 with a cone of 84V and a collision energy of 22V, Benzoyl-3-Methoxytyramine (3-MT) m/z 376.33 to m/z 105.18 with a cone of 44V and a collision energy of 16V, Benzoyl-Octopamine m/z 344.33 to m/z 105.24 and cone of 10V and collision energy of 18V. Benzoyl-Homovanillic acid (HVA) m/z 304.2 to m/z 105.23 with a cone of 2V and a collision energy of 12V, Benzoyl- 4-Aminobutyric acid (GABA) m/z 208.2 to m/z 105.18 with a cone voltage of 14V and a collision energy of 12V. All reference compounds were dissolved in water (100 µg/mL). A mix standard was prepared by using 0.5 mL of each standard in 10mL 50% Acetonitrile (5000 ng/mL), mixing 2 mL of this Mix-Standard and 1ml of 100 mM Sodium Carbonate and 1 mL 2% Benzylchloride in acetonitrile. The solution was vortexed for 30 s and 1ml of water was added before vortexing for additional 30 s and get a final concentration of 100 ng/mL. This standard was used to prepare the calibration curve with 50% acetonitrile.

PET Measurements

The PET tracers 6-[^{18}F]fluoro-L-*m*-tyrosine (6-[^{18}F]FMT) and 2-deoxy-2-[^{18}F]fluoro-D-glucose ([^{18}F]FDG) were used in this study. The mice were measured in Focus 220 micro PET scanner (CTI-Siemens) with a resolution of 1.4 mm. 6-[^{18}F]FMT PET measurements were performed with n = 6 Dnmt3a2/3L$^{Dat/wt}$ mice (4 m, 2 f) and n = 7 Dnmt3a2/3L$^{wt/wt}$ control littermates (4 m, 3 f). One hour before 6-[^{18}F]FMT injection, 15 mg/kg benserazide was administered intraperitoneally to block peripheral amino acid decarboxylase. After 45 min, mice were anesthetized with 5% isoflurane, delivered in 30% oxygen and 70% air. For maintenance of anesthesia, isoflurane concentration was reduced to 1.5–2.0%. A catheter was inserted into the lateral tail vein and the mouse was placed in an animal holder (Medres, Cologne, Germany) equipped with a respiratory mask. Body temperature was held at 37 °C by warm water flow through the animal holder. The emission scan started with i.v. 6-[^{18}F]FMT injection (9.3–17.7 MBq in 125 µL) and ended after 40 min. It was followed by a 10 min transmission scan using a ^{57}Co (cobalt-57) point source for attenuation correction. Images were reconstructed in two frames à 20 min, using an iterative OSEM3D/MAP procedure [53]. Voxel sizes were 0.38 × 0.38 × 0.82 mm^3. All further analysis was done with the Software VINCI 4.92 (MPI for Metabolism Research, Cologne, Germany). The first frame was discarded, while the second frame was co-registered manually to a mouse brain MRI template. [^{18}F]FMT uptake was normalized to occipital cortex. Dnmt3a2/3L$^{Dat/wt}$ mice were compared to controls using a *t*-test. Resulting statistical maps were corrected for multiple testing on the $p < 0.05$ level with a threshold-free cluster enhancement (TFCE) procedure [54].

For [^{18}F]FDG PET, performed with the same animals, [^{18}F]FDG (9.2–15.4 MBq in 125 µl) was injected intraperitoneally during a short (1 min) isoflurane anesthesia. The mouse was then transferred to the treadmill which was operated at low speed (0.1 m/s). After 30 min of easy running, the mouse was anestetized with isoflurane and placed in the PET scanner (see above). The emission scan started 40 min after [^{18}F]FDG injection and stopped after 30 min of data collection. It was followed by a 10 min ^{57}Co transmission scan. On a different day, the procedure was repeated and combined with home cage stay instead of treadmill running. Images were reconstructed as one frame and normalized to global mean. The whole brain volume of interest (VOI) was carefully adjusted so that the spillover from head muscles and harderian glands did not influence global mean. Treadmill and home cage conditions were compared using a paired *t*-test, the Dnmt3a2/3L$^{Dat/wt}$ and Dnmt3a2/3L$^{wt/wt}$ mice were compared using an unpaired *t*-test. Difference images "treadmill minus home cage" were correlated with 6-[^{18}F]FMT images using the Pearson correlation test. All resulting

statistical maps were corrected for multiple testing on the $p < 0.05$ level with a threshold-free cluster enhancement (TFCE) procedure [55].
